# Successful resuscitation of amniotic fluid embolism applying a new classification and management strategy

**DOI:** 10.1186/s40981-015-0001-x

**Published:** 2015-08-27

**Authors:** Shinya Yufune, Motoshi Tanaka, Ryosuke Akai, Yasushi Satoh, Kenichi Furuya, Katsuo Terui, Naohiro Kanayama, Tomiei Kazama

**Affiliations:** 1 Department of Anesthesiology, National Defense Medical College, 3-2 Namiki, Tokorozawa, Saitama 359-8513 Japan; 2 Department of Obstetrics and Gynecology, National Defense Medical College, 3-2 Namiki, Tokorozawa, Saitama 359-8513 Japan; 3 Department of Obstetric Anesthesiology, Saitama Medical Center, Saitama Medical University, 1981 Kamoda, Kawagoe, Saitama 350-8550 Japan; 4 Department of Obstetrics and Gynecology, Hamamatsu University School of Medicine, 1-20-1, Handayama, Higashi-ku, Hamamatsu, Shizuoka, 431-3192 Japan

**Keywords:** Amniotic fluid embolism, Disseminated intravascular coagulation (DIC), Uterine atony, Cesarean delivery

## Abstract

Amniotic fluid embolism (AFE) is a rare but life-threatening maternal emergency caused by the entry of amniotic fluid contents into the maternal circulation. The clinical manifestations of AFE are heterogeneous, leading to misdiagnosis or treatment delay. Kanayama and colleagues distinguished the cardiopulmonary collapse type (or classic type) from the disseminated intravascular coagulation (DIC) type of AFE on the basis of the presence of uterine atony and DIC in the latter prior to cardiopulmonary failure. We report a case of DIC-type AFE successfully treated by blood volume replacement and coagulation therapy. The patient was scheduled for elective cesarean delivery because of a previous cesarean section and moyamoya disease. Delivery was uneventful, but massive vaginal bleeding without clotting and ensuing hypovolemic shock occurred 4 h later. She was transferred to the operating room for emergency laparotomy, but sustained a cardiac arrest. The patient was successfully resuscitated and a hysterectomy performed. During surgery, the patient received fresh frozen plasma, platelets, fibrinogen, and antithrombin concentrate. In cardiopulmonary collapse type AFE, cardiopulmonary resuscitation without delay is important. In the present case of DIC-type AFE, however, early supplementation of clotting factors and platelets was critical for patient survival.

## Background

Amniotic fluid embolism (AFE), a severe maternal reaction to amniotic fluid contents entering the circulation, occurs in only 2–8 of every 100,000 deliveries [1, 2]. However, AFE has a high mortality of 21.6 % in the United States and 24.3 % in Japan [3, 4]. The risk factors associated with AFE include advanced maternal age, placental abnormalities, operative deliveries, eclampsia, polyhydramnios, cervical lacerations, and uterine rupture [5]. The three classic AFE symptoms are acute hypoxia, severe hypotension or cardiac arrest, and coagulopathy, which generally occur suddenly during labor (or pregnancy termination) or shortly after delivery [6]. However, AFE has a wide spectrum of manifestations, and patients do not always follow the classic clinical course. On the basis of this clinical heterogeneity, it was recently suggested that AFE may involve disseminated intravascular coagulation (DIC), uterine atony, and (or) cardiopulmonary collapse [4]. We present a case of AFE with cardiac arrest arising from DIC following elective cesarean delivery.

## Case presentation

A 38-year-old woman (67.3 kg, 160 cm tall, and G3 P2) with moyamoya disease was scheduled for a repeat cesarean delivery. She gave us her history of frequent transient ischemic attacks during hyperventilation associated with moyamoya disease; therefore, a cesarean delivery under general anesthesia was planned to avoid ischemic attack during labor. She was not taking any herbal preparations or anticoagulants. Laboratory findings on admission were as follows: normal electrocardiogram and chest X-ray, hemoglobin 10.7 g/dL, platelet count 32.2 × 10^4^/μL, fibrin degradation product (FDP) 6 μg/mL, D-dimer 2.2 μg/mL, fibrinogen 431 mg/dL, no urinary glucose or protein. At 38 weeks and 1 day of gestation, cesarean delivery was performed under general anesthesia without complications. The patient’s trachea was extubated and returned to the obstetrics ward.

**Fig. 1:**
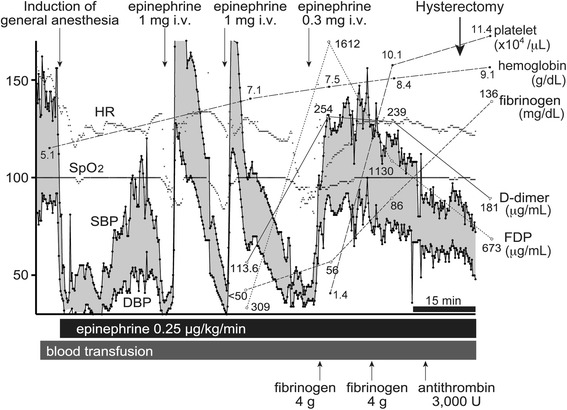
Intraoperative anesthetic course and the result of coagulation tests. *HR* heart rate (/min), *SBP* systolic blood pressure (mmHg), *DBP* diastolic blood pressure, *SpO*_2_ peripheral capillary oxygen saturation (%), *FDP* fibrin degradation products

Four hours after cesarean delivery, vaginal hemorrhage increased rapidly. The patient’s systolic arterial pressure decreased to 80 mmHg and heart rate (HR) increased to 148 beats/min. Ultrasound imaging and computed tomography (CT) revealed a large retrovesical hematoma. The patient was transferred to the operating room for emergency laparotomy, and general anesthesia was induced. A few minutes after skin incision, airway pressure increased suddenly, SpO_2_ declined gradually, arterial blood pressure became undetectable, and HR was absent. Immediate cardiopulmonary resuscitation was initiated. Spontaneous circulation resumed after administration of 1 mg epinephrine, but additional bolus injections and continuous epinephrine infusion were required to maintain spontaneous circulation. The uterus was atonic and swollen to the size of a person’s head. Although a B-Lynch suture was attempted, atonic hemorrhage remained uncontrolled and the decision to perform hysterectomy was made. A large amount of bloody fluid was obtained from the surgical site, and the blood did not clot. Bleeding from the nose, the oral cavity, and the tracheal tube was observed. Coagulation tests, including those for FDP, D-dimer, and fibrinogen, showed that fibrinogen had decreased to <50 mg/dL. We administered 44 units of packed red blood cells (PRBCs), 44 units of fresh frozen plasma (FFP), 55 units of platelets, 8 g of fibrinogen, and 3,000 international units (IU) of antithrombin concentrate during the surgery (In Japan, estimated volume per unit of blood products is as follows: PRBCs 140 mL/unit, FFP 120 mL/unit, platelet 20 mL/unit). Intraoperative anesthetic course and the result of coagulation tests are shown in Fig. 1. After hemodynamic conditions stabilized, hemorrhage from the nose, the oral cavity, and the tracheal tube gradually decreased, and the patient was transferred to the intensive care unit (ICU) without extubation.

Computed tomography after surgery revealed pulmonary embolism (PE) but no cerebral hemorrhage. Heparin therapy was initiated to maintain activated partial thromboplastin time (APTT) within the therapeutic target range of 50–70 s. Results of intraoperative maternal serum analysis were as follows: zinc coproporphyrin-1 (Zn-CP1) of 4 pmol/mL (normal, <1.6 pmol/mL), sialyl-Tn antigen (STN) 20.0 IU/mL (normal, <46 IU/mL), compliment factor 3 (C3) 58 mg/dL (normal, 80–140 mg/dL), C4 9 mg/dL (normal, 11–34 mg/dL), and interleukin-8 (IL-8) 405 pg/mL (normal, <20 pg/mL). Increased Zn-CP1 and IL-8 with decreased C3 and C4 strongly suggested AFE [7].

**Fig. 2:**
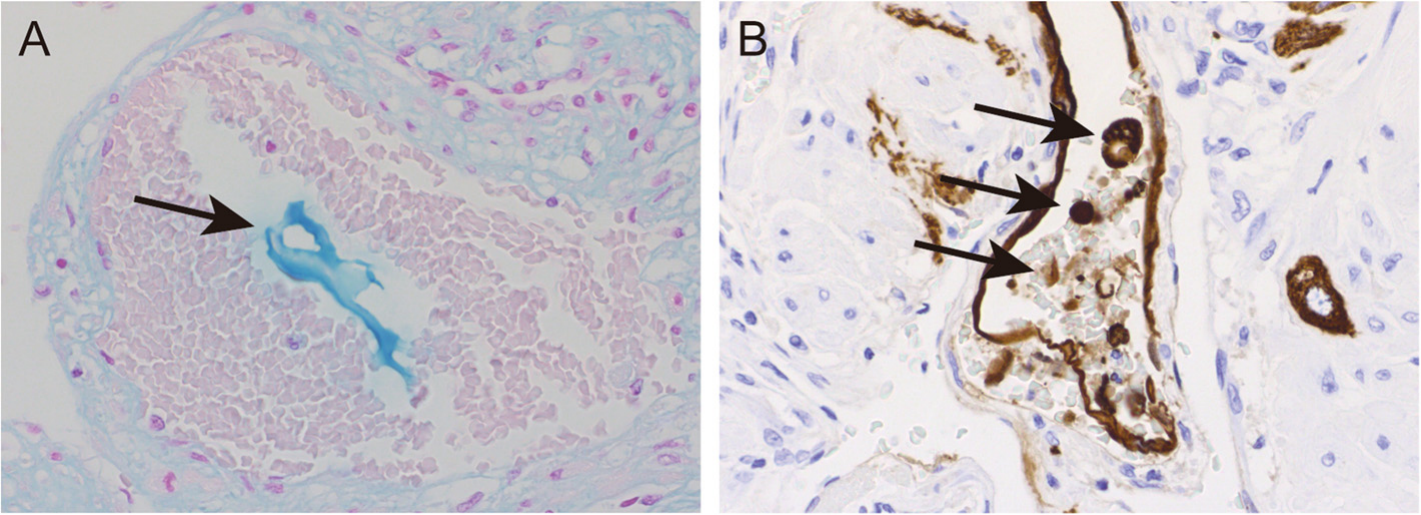
Histological sections of the uterus. **a** Acid mucopolysaccharidic matter was observed in uterine vessels (*black arrow*), Alcian blue staining, 20×. **b** Immunohistochemically marked epithelial squames in uterine vessels (*black arrows*), Cytokeratin AE1/AE3 immunohistochemical staining, 20×

The pathological findings from the uterus of this patient are shown in Fig. 2. The amniotic fluid-derived ingredients are not detected in the hematoxylin-eosin (HE) staining, but Anti-Pan Cytokeratin (AE1/AE3) stains detected amniotic fluid-derived fetal keratin (Fig. 2a) and alcian blue staining detected amniotic fluid-derived mucin (Fig. 2b) and edematous changes are observed in the uterus.

Seven days after the surgery, the patient’s trachea was extubated and discharged from the ICU. Anticoagulant therapy was switched from heparin infusion to warfarin. Eighteen days after surgery, she was discharged from the hospital.

### Discussion

Steiner and Lushbaugh (1941) first reported the cases of eight women who died unexpectedly from “obstetric shock” associated with fetal material in pulmonary vessels [8]. They concluded that pulmonary embolism from particulate matter in amniotic fluid was the most likely cause of death. Since then, many investigators have tried to clarify the pathophysiology of AFE, but the etiology remains controversial. Recent studies suggest that, in addition to mechanical obstruction of pulmonary vessels, AFE can also result from a systemic inflammatory response syndrome (SIRS), manifesting as anaphylaxis or septic shock [2]. Thus, Clark suggests the term “anaphylactoid syndrome of pregnancy” to better describe the pathophysiology of AFE [6].

The clinical manifestations of AFE include sudden hypoxia, severe hypotension (including cardiac arrest), and DIC. In the classic AFE type, acute dyspnea and desaturation appear during labor or shortly after delivery, followed by cardiopulmonary collapse and coagulopathy. However, we have observed many cases of *forme fruste* AFE in which one or more components of this symptom triad were minimal or absent. Kanayama et al. suggested that AFE can be separated into cardiopulmonary collapse and DIC types on the basis of clinical findings (Table 1) [4, 7]. In their report, the autopsies of five cardiopulmonary collapse-type patients revealed no remarkable changes in the uteri, whereas examination of four DIC-type patients showed amniotic components in all uteri, suggesting that the reaction of amniotic components to the uterus can cause uterine atony and DIC [7]. In typical DIC-type AFE, the patient shows uterine atony followed by DIC prior to cardiopulmonary symptoms; therefore, volume resuscitation and the supplementation of platelets and clotting factors are essential to save such patients.

**Table 1:** New classification of amniotic fluid embolism

	Initial symptoms	Time from symptom onset to cardiac arrest	Histology	Initial management
Cardiopulmonary collapse type (Classic type)	• Sudden dyspnea	Very short (0–60 min in typical cases)	• Amniotic components in pulmonary vessels	Cardiopulmonary resuscitation including inotropes
	• Severe hypotension (including cardiac arrest)			
	• Seizure			
DIC type	• Massive bleeding without clotting	Several hours	• Amniotic components in uterus and/or uterine vessels	Volume resuscitation including supplement of platelets and clotting factors
	• Uterine atony		• Thrombus in uterine vessels	
			• Intestinal edema in uterus	

Modified from [7]

*DIC* disseminated intravascular coagulation

To manage hemorrhage during surgery, transfusion of PRBCs is recommended to maintain oxygen transport at first, and coagulation tests and platelet count should be obtained before administering FFP and platelets [9]. However, many AFE patients require immediate FFP and platelet transfusion as well as packed red cells due to the consumption of coagulation factors at the early stage of AFE. The blood transfusion strategy for AFE patients is thus similar to the massive transfusion protocol for trauma patients, in which disruption of hemostasis occurs because of dilution coagulopathy, inflammatory mediator activation, hyperfibriolysis, thrombocytopenia, and metabolic abnormalities [10]. In our case, we recognized that vaginal bleeding without clotting was an early sign of DIC-type AFE and promptly initiated transfusion of PRBCs and FFP (1:1), platelets, fibrinogen, and antithrombin concentrate.

The diagnosis of AFE is based primarily on clinical observation. In the past, the detection of fetal squamous cells in the maternal pulmonary circulation was considered a definitive diagnostic finding of AFE. However, a recent study detected fetal squamous cells in only 50 % AFE patients during the aspiration of pulmonary artery blood [6], whereas another report found that the presence of squamous cells in the maternal circulation was not always associated with AFE [11]. Although there is no gold standard diagnostic marker for AFE, we believe that changes in a number of serum factors are strongly indicative of AFE, including Zn-CP1, STN, C3, C4, and IL-8. Meconium is rich in Zn-CP1 [12], and STN is a sugar chain of the mucin-type glycoprotein that recognizes mucin in the meconium/amniotic fluid [13]. We thus propose that STN and Zn-CP1 in the maternal circulation are suggestive of AFE [7]. Benson et al. reported that C3 and C4 levels in AFE patients were significantly lower than normal [14], suggesting that complement activation has an important role in the pathophysiology of AFE. Amniotic fluid components can also activate immune cells associated with allergic reactions, which produce large amounts of bradykinin and inflammatory cytokines such as IL-8 [7]. Recently, C1 estarase inhibitor activity (C1INH) was suggested as a prognostic marker of AFE [15]. In addition to serum factors, histological observation revealed edematous changes exist in the uterine smooth muscle and amniotic fluid components and fetal epithelial cells in the uterine vessels in this patient are suggestive of DIC-type AFE [7].

In this particular case patient, the reasons for cardiac arrest are unclear. The possibilities include superimposed cardiopulmonary type AFE and pulmonary thromboembolism. Although the patient showed no symptoms of deep vein thrombosis (DVT) before cesarean delivery, preoperative D-dimer levels were elevated; hence, DVT is possible. Interestingly, both serum FDP and D-dimer decreased promptly after administration of fibrinogen and antithrombin concentrate. The onset of cardiac arrest was more than 4 h after delivery, not sudden, and initial symptoms was massive bleeding without clotting, we considered cardiac arrest was due to hypovolemic shock. We cannot entirely exclude the classic type of AFE as the causation of cardiac arrest, although initial manifestation, change of serum factors, and histological observations in uterus are suggestive of DIC-type AFE. Appropriate treatment for DIC at the right time might lead to her recovery from DIC.

## Conclusions

In conclusion, we describe a case of DIC-type AFE. Unlike cardiopulmonary type AFE, uterine atony and DIC were the initial symptoms. Early diagnosis and prompt administration of blood products, including clotting factors and platelets, are essential for patient recovery.

## Consent

Written informed consent was obtained from the patient for publication of this case report and any accompanying images. A copy of the written consent is available for review by the Editor-in-chief of this journal.

## Abbreviations

AFE: amniotic fluid embolism
APTT: activated partial thromboplastin time
C1INH: C1 estarase inhibitor activity
C3: compliment factor 3
C4: compliment factor 4
CT: computed tomography
DBP: diastolic blood pressure
DIC: disseminated intravascular coagulation
DVT: deep vein thrombosis
FDP: fibrin degradation products
FFP: fresh frozen plasma
HE: hematoxylin-eosin
HR: heart rate
ICU: intensive care unit
IL-8: interleukin-8
IU: international units
PE: pulmonary embolism
PRBCs: packed red blood cells
SBP: systolic blood pressure
SIRS: systemic inflammatory response syndrome
SpO_2_: peripheral cappilary oxygen saturation
STN: sialyl-Tn antigen
Zn-CP1: zinc coproporphyrin-1
